# Supplementary material for: Effect of Different Drying Methods on the Quality of Oudemansiella raphanipes
Source: Foods. 2024 Apr 1;13(7):1087. doi: 10.3390/foods13071087 (PMC11011357; doi:10.3390/foods13071087)
Supplement: Supplementary file 1 [file foods-13-01087-s001.zip › foods-2897526-supplementary materials.pdf]

A

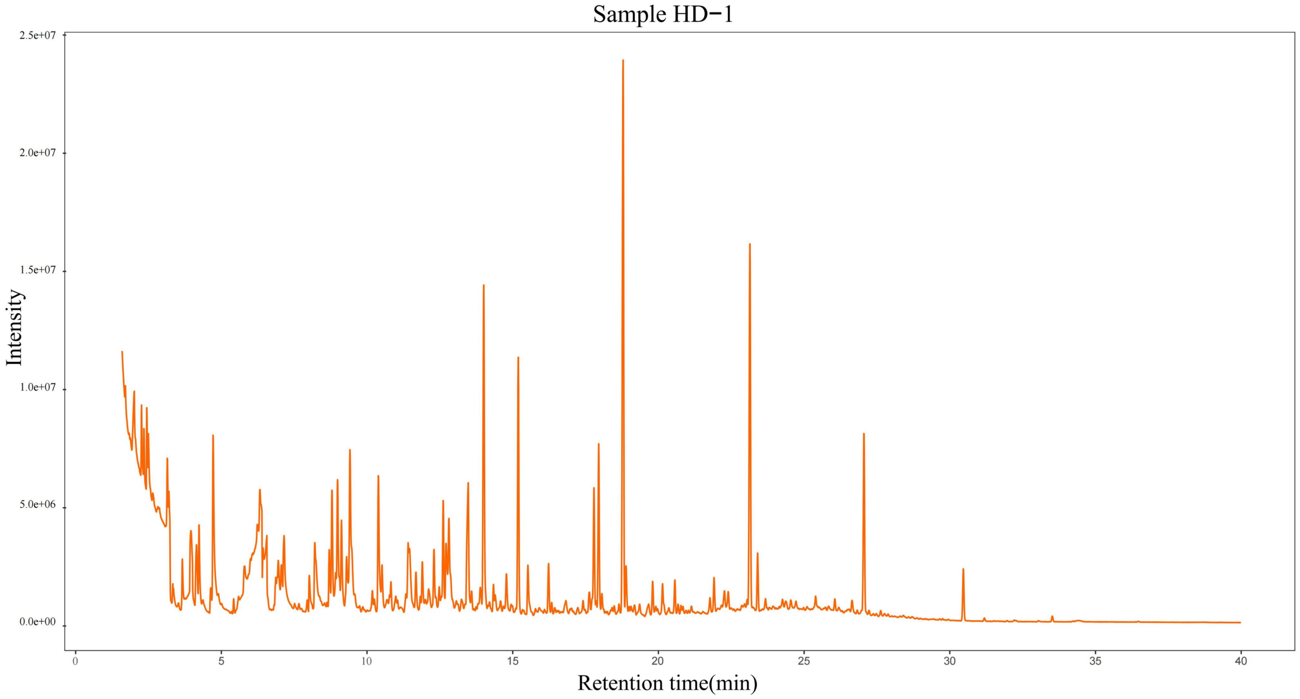
Sample HD−1
Intensity
2.5e+07
2.0e+07
1.5e+07
1.0e+07
5.0e+06
0.0e+00
0
5
10
15
20
25
30
35
40
Retention time(min)

B

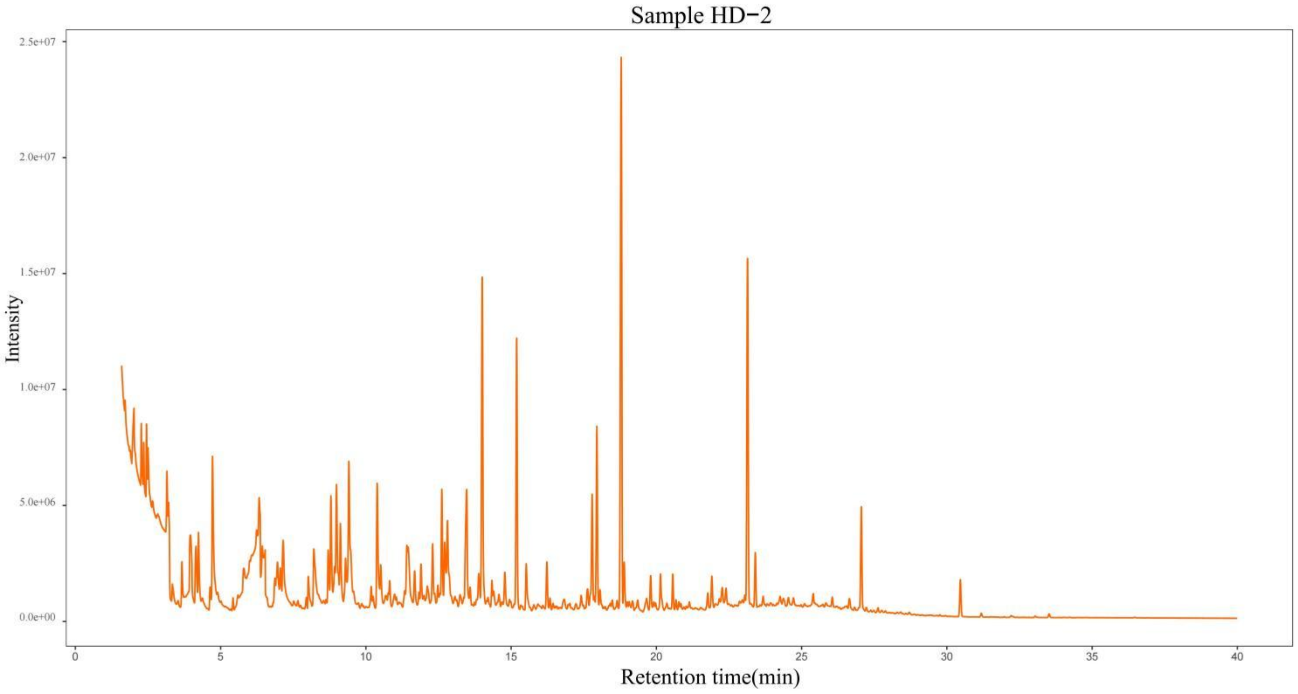
Sample HD−2
Intensity
2.5e+07
2.0e+07
1.5e+07
1.0e+07
5.0e+06
0.0e+00
0
5
10
15
20
25
30
35
40
Retention time(min)

C

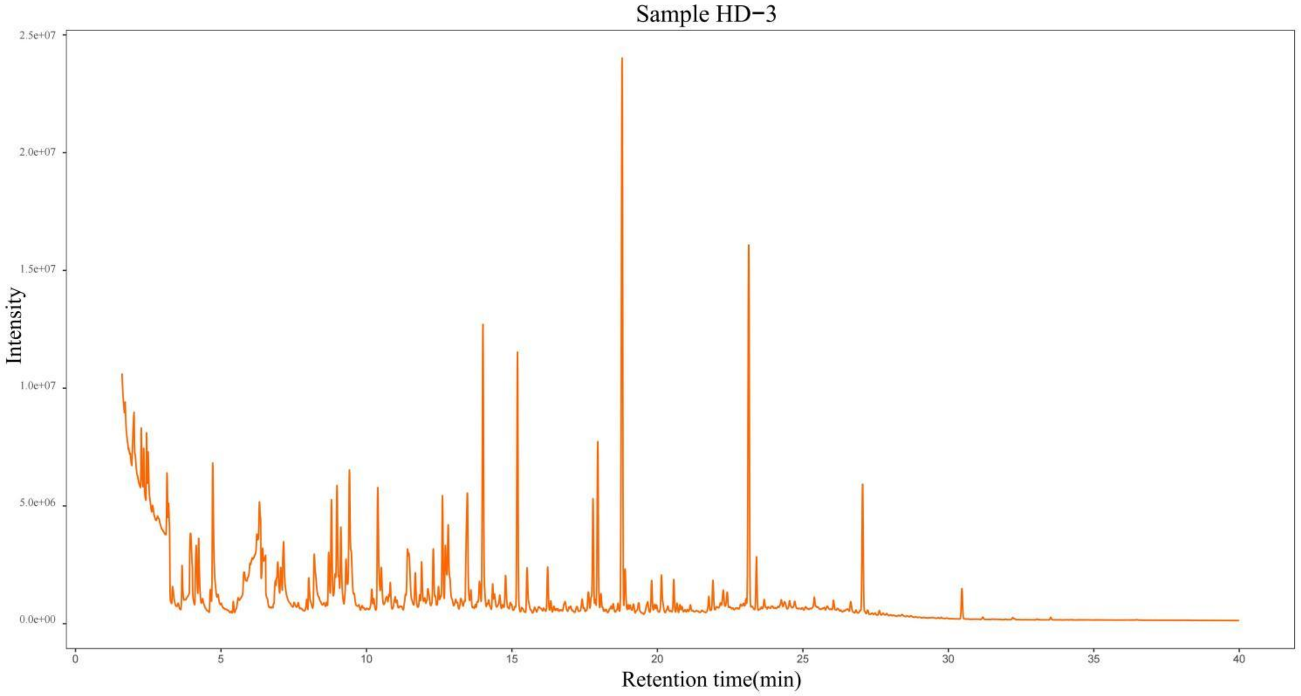
Sample HD−3
2.5e+07
2.0e+07
1.5e+07
1.0e+07
5.0e+06
0.0e+00
Intensity
0
5
10
15
20
25
30
35
40
Retention time(min)

D

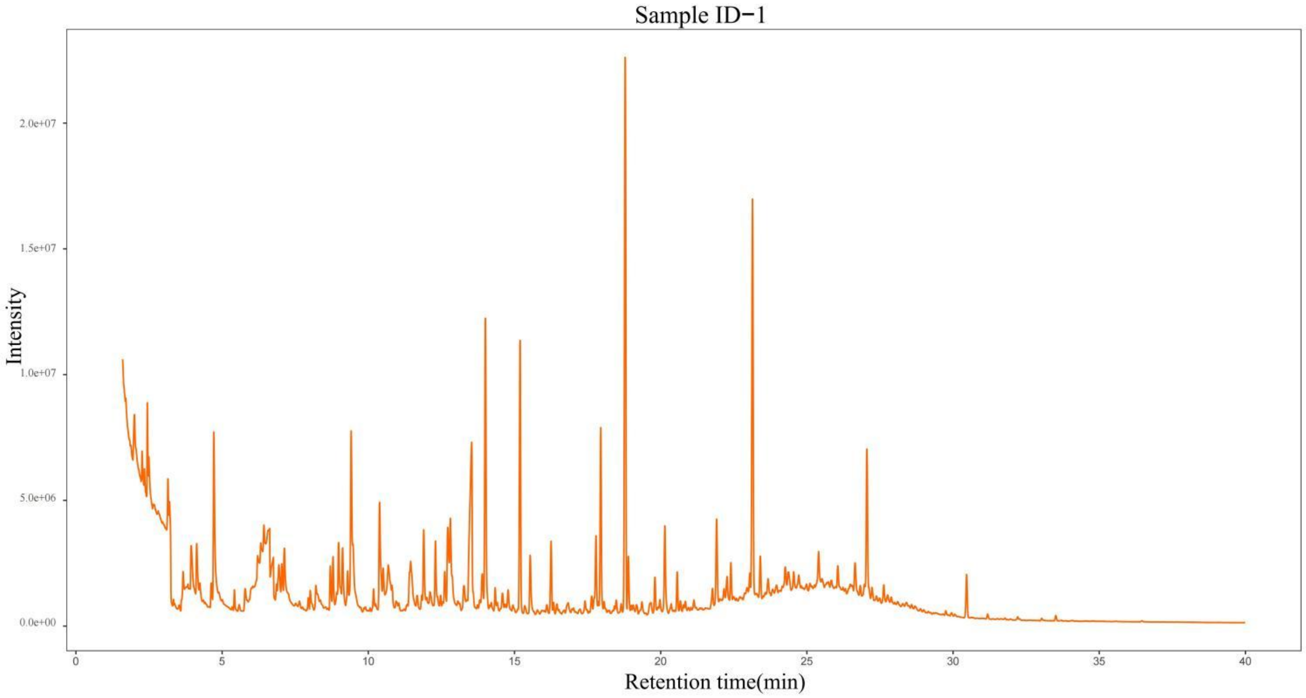
Sample ID−1
2.0e+07
1.5e+07
1.0e+07
5.0e+06
0.0e+00
Intensity
0
5
10
15
20
25
30
35
40
Retention time(min)

E

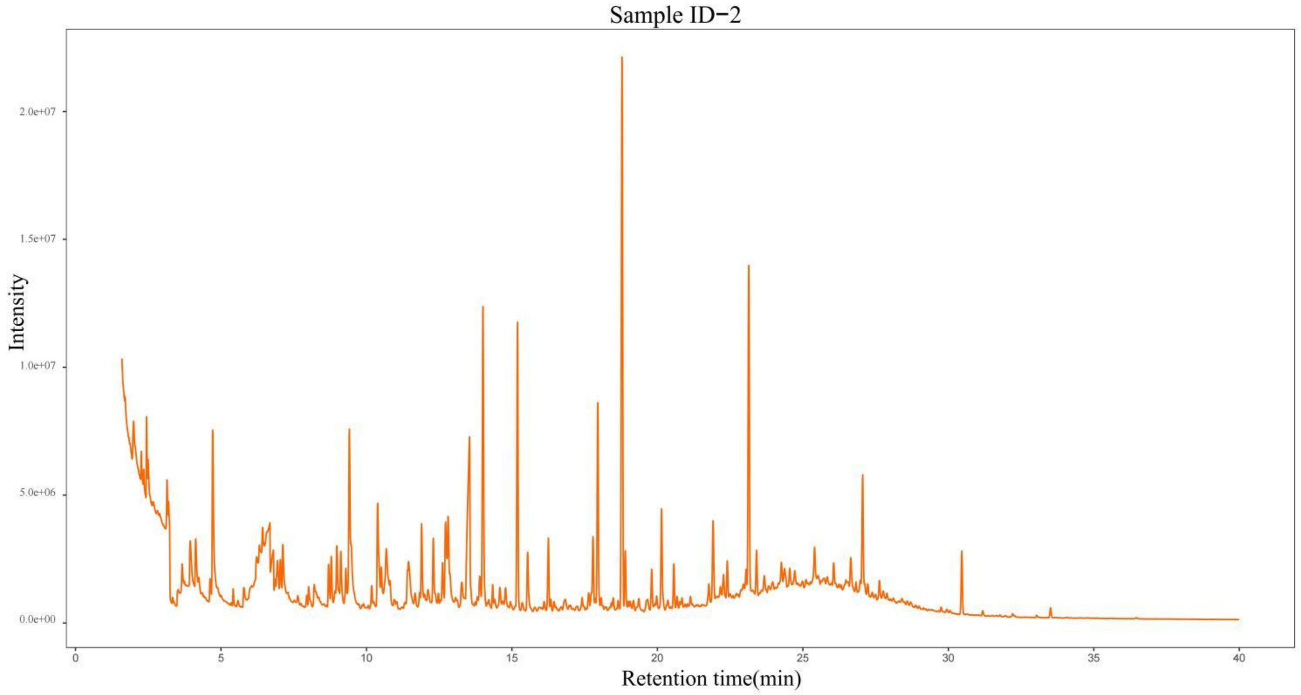
Sample ID−2
Intensity
2.0e+07
1.5e+07
1.0e+07
5.0e+06
0.0e+00
0
5
10
15
20
25
30
35
40
Retention time(min)

F

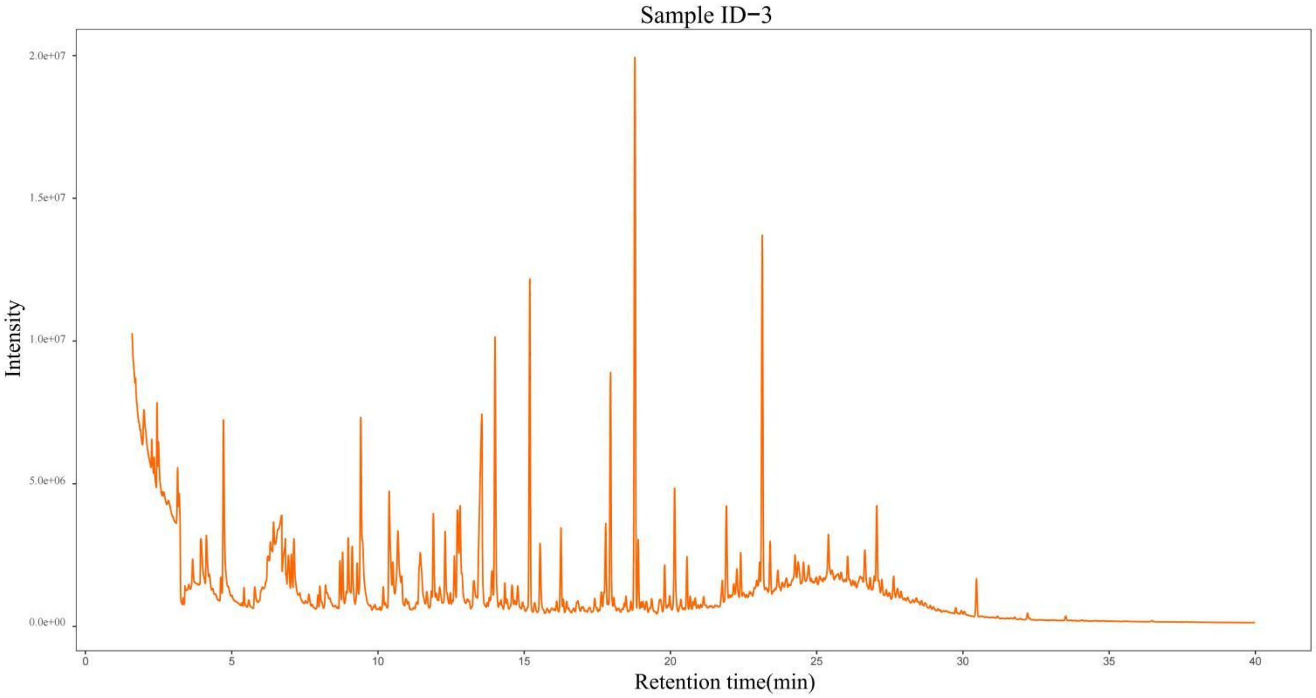
Sample ID−3
Intensity
2.0e+07
1.5e+07
1.0e+07
5.0e+06
0.0e+00
0
5
10
15
20
25
30
35
40
Retention time(min)

G

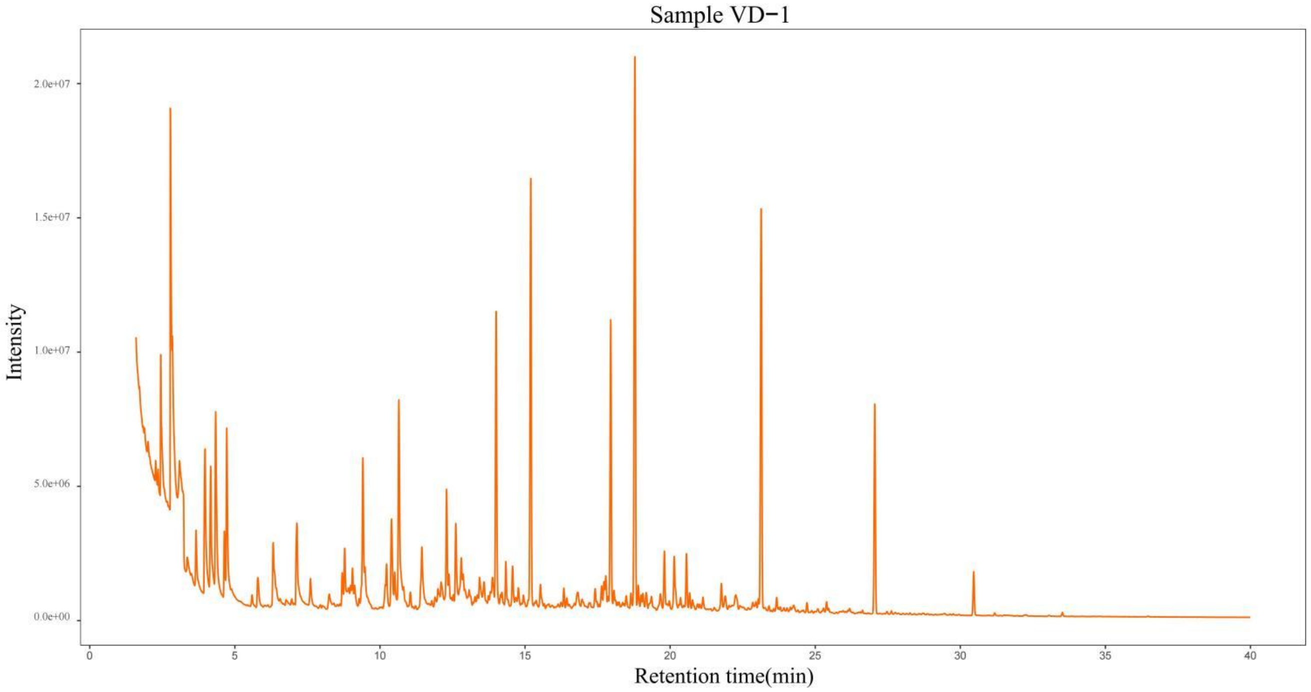

Sample VD-1
Intensity
2.0e+07
1.5e+07
1.0e+07
5.0e+06
0.0e+00
0
5
10
15
20
25
30
35
40
Retention time(min)

H

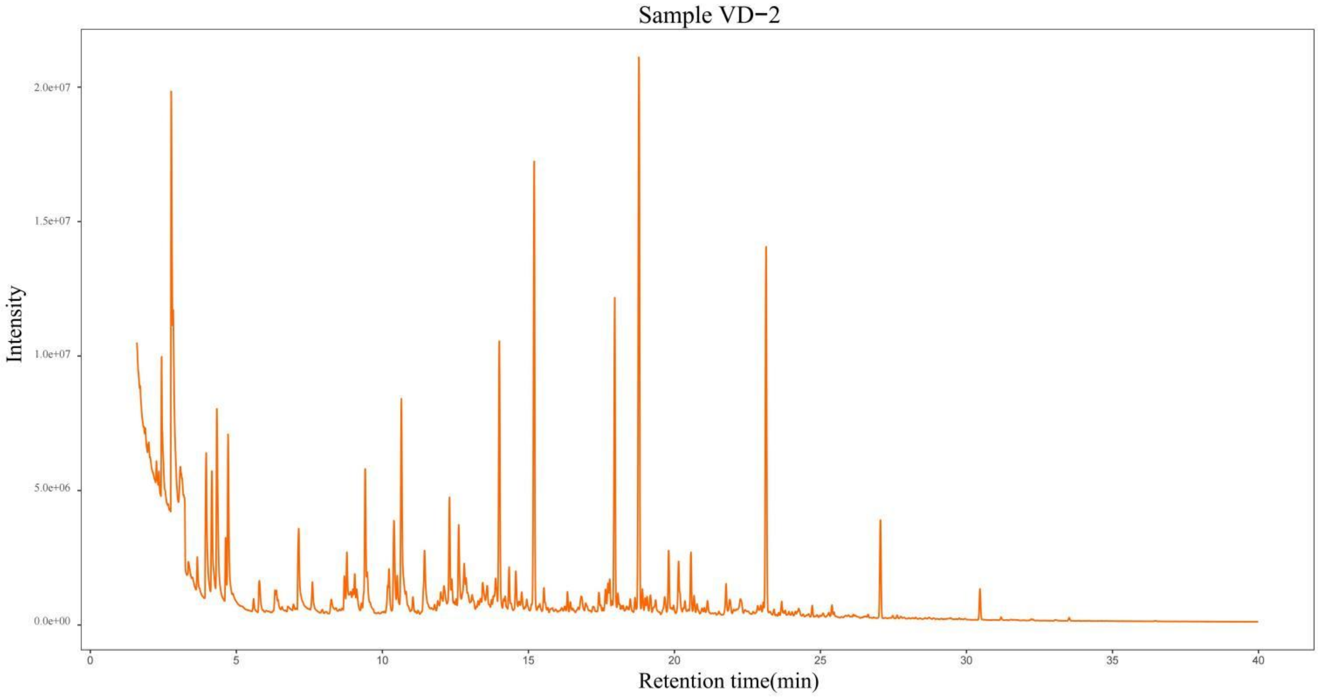

Sample VD-2
Intensity
2.0e+07
1.5e+07
1.0e+07
5.0e+06
0.0e+00
0
5
10
15
20
25
30
35
40
Retention time(min)

I

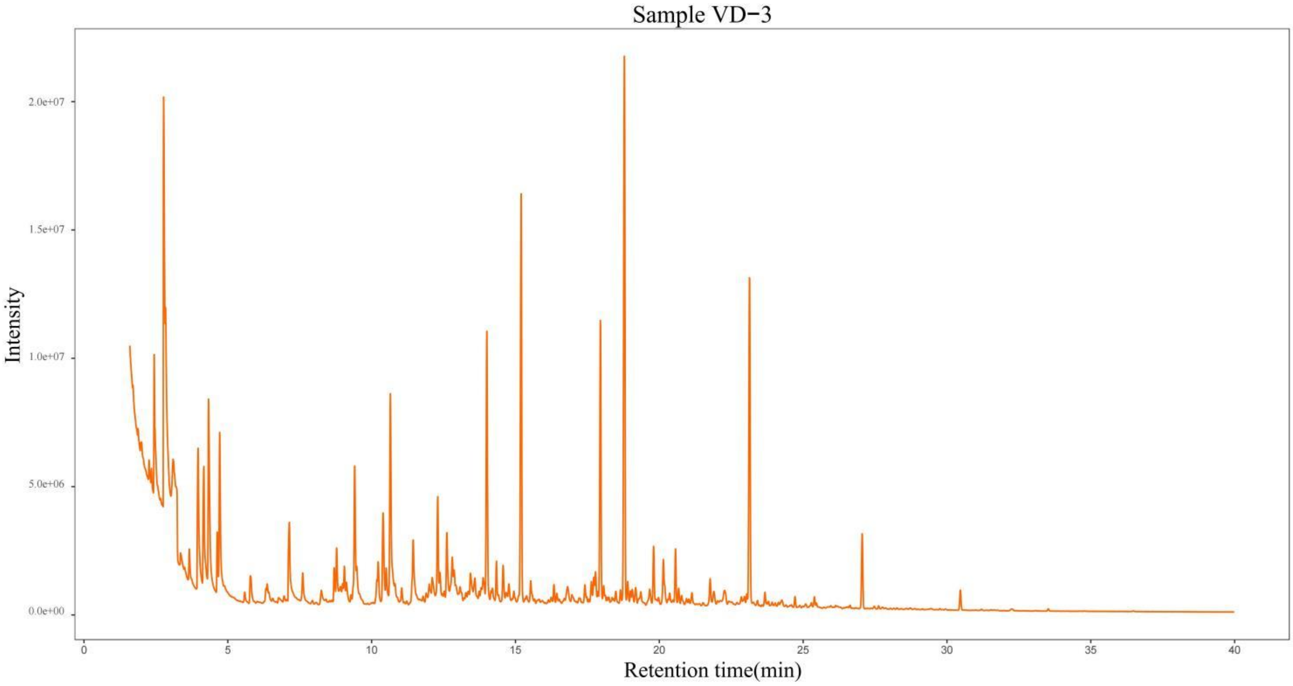

Figure S1 Total Ion Chromatogram of volatile compounds in *O. raphanipes* dried using different methods,(A-C)HD-treated samples, (D-F)ID-treated samples, (G-I)VD-treated samples.
